# Illinois State Medical Society

**Published:** 1857-07

**Authors:** 


*Illinois State Medical Society.*

The regular annual meeting of this Society was held in the medical college building in this city, on the 2d, 3d, and 4th days of June.

The meeting was well attended, there being about forty members present. The time was diligently and profitably occupied in the reading and discussion of important reports and papers. The address of the President, Dr. H. Noble, was listened to with much interest. The following were the principal reports and papers presented, viz.:

Report on Practical Medicine and Epidemics, by Dr. C. N. Andrews, of Rockford.

Report on Drugs and Medicines, by Dr. H. A. Johnson, of Chicago.

Report on the Changes produced in the Blood in Continued Fevers, by Dr. N. S. Davis, of Chicago.

Report on the Medicinal Properties of the Asclepias Tuberosa, by Dr. C. Goodbrake, of Clinton.

A Paper on the Stomatitis Materni, by Dr. W. M. Chambers, of Charleston.

Report on Congestive Intermittents, by Dr. F. K. Bailey, of Joliet.

A Paper on Ununited Fractures, by Dr. D. Brainard, of Chicago.

Dr. J. W. Freer, of Chicago, exhibited to the Society an interesting pathological specimen, consisting of an encysted disease of the lower end of the femur.

The following officers were chosen for the ensuing year:

*For President*, Dr. C. Goodbrake, of Clinton.

*Vice-Presidents*, Drs. A. L. McArthur, of Joliet, W. M. Chambers, of Charleston.

*Permanent Secretary*, Dr. H. A. Johnson, of Chicago.

*Assistant Secretary*, Dr. C. N. Andrews, of Rockford.

This much we have given from memory. A fuller account of the proceedings, and a list of the committees appointed, will be given when we get an official copy of the proceedings from the Secretary.

Rockford was selected as the place for the next annual meeting of the Society.

